# Doppler Flow Response Following Running Exercise Differs Between Healthy and Tendinopathic Achilles Tendons

**DOI:** 10.3389/fphys.2021.650507

**Published:** 2021-03-23

**Authors:** Lucie Risch, Frank Mayer, Michael Cassel

**Affiliations:** ^1^University Outpatient Clinic, Sports Medicine and Orthopedics, University of Potsdam, Potsdam, Germany; ^2^Faculty of Health Sciences, University of Potsdam, Potsdam, Germany

**Keywords:** neovascularization, tendinopathy, Doppler ultrasound, Advanced Dynamic Flow, athlete, sonography

## Abstract

**Background:**

The relationship between exercise-induced intratendinous blood flow (IBF) and tendon pathology or training exposure is unclear.

**Objective:**

This study investigates the acute effect of running exercise on sonographic detectable IBF in healthy and tendinopathic Achilles tendons (ATs) of runners and recreational participants.

**Methods:**

48 participants (43 ± 13 years, 176 ± 9 cm, 75 ± 11 kg) performed a standardized submaximal 30-min constant load treadmill run with Doppler ultrasound “Advanced dynamic flow” examinations before (U_pre_) and 5, 30, 60, and 120 min (U_5_-U_120_) afterward. Included were runners (>30 km/week) and recreational participants (<10 km/week) with healthy (H_run_, *n* = 10; H_rec_, *n* = 15) or tendinopathic (T_run_, *n* = 13; T_rec_, *n* = 10) ATs. IBF was assessed by counting number [n] of intratendinous vessels. IBF data are presented descriptively (%, median [minimum to maximum range] for baseline-IBF and IBF-difference post-exercise). Statistical differences for group and time point IBF and IBF changes were analyzed with Friedman and Kruskal-Wallis ANOVA (α = 0.05).

**Results:**

At baseline, IBF was detected in 40% (3 [1–6]) of H_run_, in 53% (4 [1–5]) of H_rec_, in 85% (3 [1–25]) of T_run_, and 70% (10 [2–30]) of T_rec_. At U_5_ IBF responded to exercise in 30% (3 [−1–9]) of H_run_, in 53% (4 [−2–6]) of H_rec_, in 70% (4 [−10–10]) of T_run_, and in 80% (5 [1–10]) of T_rec_. While IBF in 80% of healthy responding ATs returned to baseline at U_30_, IBF remained elevated until U_120_ in 60% of tendinopathic ATs. Within groups, IBF changes from U_pre_-U_120_ were significant for H_rec_ (*p* < 0.01), T_run_ (*p* = 0.05), and T_rec_ (*p* < 0.01). Between groups, IBF changes in consecutive examinations were not significantly different (*p* > 0.05) but IBF-level was significantly higher at all measurement time points in tendinopathic versus healthy ATs (*p* < 0.05).

**Conclusion:**

Irrespective of training status and tendon pathology, running leads to an immediate increase of IBF in responding tendons. This increase occurs shortly in healthy and prolonged in tendinopathic ATs. Training exposure does not alter IBF occurrence, but IBF level is elevated in tendon pathology. While an immediate exercise-induced IBF increase is a physiological response, prolonged IBF is considered a pathological finding associated with Achilles tendinopathy.

## Introduction

Intratendinous blood flow (IBF) and degenerative tendon changes are a common finding in long-distance runners with Achilles tendinopathy (Hirschmüller et al., 2010). In earlier studies IBF has been associated with ingrowth of neo-innervation in a failed tendon healing process and the onset of tendon pain (Alfredson et al., 2003; Rees et al., 2014). More recent studies, however, have reported no direct association of IBF with pain severity or functional impairment (de Vos et al., 2007; De Jonge et al., 2014; De Marchi et al., 2018). By use of high-resolution ultrasound devices and increasing sensitivity to low flow (Koenig et al., 2010; Risch et al., 2016), IBF has also become detectable in up to 35% of asymptomatic Achilles tendons (ATs) (Boesen et al., 2006b; Hirschmüller et al., 2010). A small amount of IBF at rest has been suggested to be of physiological nature (Boesen et al., 2006a,b, 2012). Moreover, IBF has been found to increase in response to tendon loading exercise (Boesen et al., 2006a,b; Fahlström and Alfredson, 2010; Sanz-López et al., 2016; Risch et al., 2018a) assuming a physiological metabolic reaction visualized due to increased flow velocity and/or volume in pre-existing vessels (Boesen et al., 2006a,b, 2012; Risch et al., 2018a). In summary, detectability of IBF itself cannot be considered a pathological finding anymore (Boesen et al., 2006b, 2012; Risch et al., 2018a).

In athletes, several studies have reported an exercise-induced increase of IBF suggesting an adaptation to continuous high strain and mechanical loading (Boesen et al., 2006a, 2012; Malliaras et al., 2008) associated with increased metabolic activity and regeneration (remodeling/repair) (Malliaras et al., 2008; Koenig et al., 2010; Tardioli et al., 2012). This increased metabolic response, has been already reported in the context of elevated collagen synthesis and tendon hypertrophy following strenuous activity (Couppe et al., 2008; Boesen et al., 2011). Increased presence of IBF has been associated with greater hours of training and higher amount of training years, suggesting a long-term adaptation to tendon loading in athletes (Malliaras et al., 2008; Boesen et al., 2012). Then again, the amount of training hours has also been associated with an increased risk of developing Achilles tendinopathy (Knobloch et al., 2008). An acute increase in amount of sonographic detectable intratendinous vessels following exercise has been suggested to represent elevated blood flow in preexisting vessel beds, rising above the sensitivity threshold of Doppler ultrasound (Boesen et al., 2006b; Risch et al., 2018a). Other studies, however, have found contradictory effects of exercise on detectable IBF in terms of persisting, decreased (Malliaras et al., 2012) or absent blood flow (Pingel et al., 2013b). Due to varying sensitivity and imaging quality of ultrasound devices (Boesen et al., 2012), lacking standardization of exercise and examination protocols as well as inconsistent tendon characterization (healthy, tendinopathy, degenerative changes), comparability of previous studies and interpretation of differing results is limited (Risch et al., 2018a). Consequently, the diagnostic value of sonographic detectable IBF considering a pathological or physiological finding remains inconclusive. To differentiate between normal and abnormal Doppler flow, a cut-off value of 1–2 vessels has been proposed, but so far lacks sufficient evidence (Koenig et al., 2010; Boesen et al., 2012).

It remains questionable to what extent training status as well as healthy or pathological tendon condition and symptoms affect detectability of exercise-induced IBF. Therefore, the aim of this study was to investigate the response of sonographic detectable IBF to a standardized acute bout of constant load running exercise in healthy and tendinopathic ATs of runners and recreational participants. This study is the first to examine the acute sonographic detectable IBF response as well as IBF recovery in subsequent ultrasound examinations up to 2 h following exercise. It was hypothesized that exercise leads to an acute, temporary increase of IBF in healthy and tendinopathic tendons. Due to the higher training exposure this response was expected to be more pronounced in runners compared to recreational participants.

## Materials and Methods

### Study Design

**FIGURE 1:**
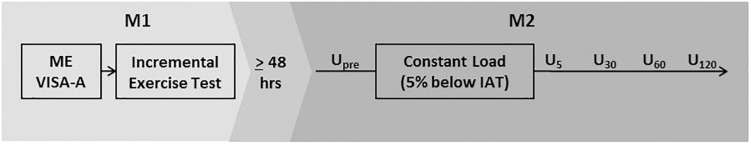
Study design. ME, medical examination; VISA-A, Victorian Institute of Sports Assessment-Achilles Questionnaire; U_pre_-U_120_, Ultrasound examinations at baseline and 5, 30, 60, and 120 min after exercise; IAT, individual anaerobic threshold.

In this cross-sectional study, all participants were assessed on two measurement days (M1/M2) separated by at least 48 h. M1 consisted of a maximum incremental treadmill running test to determine the individual anaerobic threshold (IAT). On M2 participants performed 30 min of constant load treadmill running with an intensity 5% below the IAT, and Doppler ultrasound (DU) examinations directly before (U_pre_) and 5, 30, 60, and 120 min after exercise (U_5_, U_30_, U_60_, U_120_, Figure 1). The study protocol has been validated in a prior pilot study (Risch et al., 2018a) and was approved by the local ethics committee.

### Participants

**FIGURE 2:**
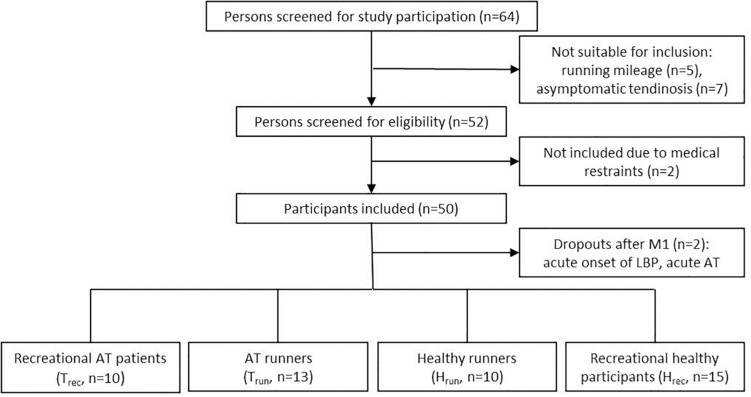
Study flow chart.

Fifty participants were included in this study between August 2015 and October 2018 after signing a written informed consent. Two participants dropped out of the study after M1, one healthy recreational participant due to an acute onset of low back pain and one recreational participant with tendinopathy due to a recurrent onset of intense Achilles tendon pain. Finally, forty-eight participants (43 ± 13 years, 176 ± 9 cm, 75 ± 11 kg) were included in the analysis. Participants were recruited from the university outpatient clinic and a local running club. Inclusion criteria were 30 to 65 years of age, with (a) presence of clinically diagnosed mid-portion Achilles tendinopathy and sonographic evident degenerative changes (focal thickening or hypo- and/or hyperechogenic areas) with a running minimum of 30 km/week (Clermont et al., 2019) (T_run_, *n* = 13) or being recreationally active (<10 km running/week, T_rec_, *n* = 10) or (b) healthy, asymptomatic ATs (no acute or past history of AT pain or sonographic signs of tendon pathology) with a running minimum of 30 km/week (H_run_, *n* = 10) or being recreationally active (<10 km running/week, maximum of two recreational training session/week, H_rec_, *n* = 15) (Figure 2 and Table 1). Patients with a history of systemic diseases (e.g., cardiovascular, metabolic or rheumatic disease, hypercholesterolemia), previous AT ruptures (partial or complete tear), previous injections (i.e., corticosteroid or platelet-rich plasma), or surgical interventions of the AT were excluded from the study. Prior to the first measurement all participants received a medical examination by a sports medicine physician (medical history and orthopedic physical examination, resting-ECG) as well as an ultrasound examination of both ATs to ensure correct inclusion and group allocation. Groups were matched for age and gender to account for the evident effect on presence of IBF and Achilles tendinopathy (Boesen et al., 2006b; Malliaras et al., 2012; Wezenbeek et al., 2018). All participants had to avoid physical activity 24 h prior to each measurement to prevent an effect of exercise on the baseline examination (de Vos et al., 2007; Sengkerij et al., 2009). Furthermore, all participants completed the Victorian Institute of Sports Assessment-Achilles (VISA-A) questionnaire to assess severity of Achilles tendinopathy (Robinson et al., 2001).

**TABLE 1:** Participants’ characteristics (mean ± SD) *n* = 48, (*n* = 2 drop-outs).

Groups	Gender (f/m)	Age (yrs)	Height (cm)	Weight (kg)	Running* (km/wk)	VISA-A* (n/100)	Tendon* thickness (a-p)	CL velocity* (km/h)
T_run_	3/10	41 ± 15	177 ± 10	73 ± 11	55 ± 14	76 ± 12	6.8 ± 1.1	12.7 ± 2.2
T_rec_	3/7	47 ± 12	175 ± 12	76 ± 12	5 ± 5	76 ± 20	7.0 ± 1.8	9.3 ± 2.2
H_run_	2/8	39 ± 10	178 ± 8	75 ± 12	57 ± 16	100 ± 0	5.5 ± 0.6	11.4 ± 1.3
H_rec_	5/10	43 ± 13	175 ± 8	75 ± 9	3 ± 4	100 ± 0	5.0 ± 0.8	9.3 ± 2.3

*Significant group differences: Running km/week (*p* < 0.01): H_rec_ vs. H_*run*_ and T_run_ (*p* < 0.01), and T_rec_ vs. H_*run*_ and T_run_ (*p* > 0.01); VISA-A (*p* < 0.01): H_rec_ vs. T_run_ and T_rec_ (*p* < 0.01), and H_*run*_ vs. T_run_ and T_rec_ (*p* < 0.01); tendon thickness (*p* < 0.01): H_rec_ vs. T_run_ and T_rec_ (*p* < 0.01), and H_*run*_ vs. T_run_ (*p* = 0.04) and T_rec_ (*p* = 0.02); CL (constant load) velocity (*p* < 0.01): H_rec_ vs. T_run_ (*p* < 0.01), T_run_ vs. T_rec_ (*p* < 0.01).

### Exercise Protocol

All running tasks were performed on the same treadmill (Pulsar, h/p/cosmos Sports & Medical, Nussdorf-Traunstein, Germany) with a constant incline of 0.4%, validated to be comparable to outdoor running (Mugele et al., 2018). To determine the IAT on M1, participants performed a stepwise incremental exercise test starting with 4–6 km/h with increments of 1–2 km/h every 3 min until voluntary exhaustion. Starting velocity and increase of stepwise increments (1 or 2 km/h) were adapted to the participants estimated performance capacity. Blood lactate was assessed by taking blood samples from the earlobe at baseline and every 3 min during the test. Heart rate was assessed simultaneously with a chest strap. IAT was determined using the Dickhuth threshold concept (Dickhuth et al., 1999). On M2, participants performed a 30 min constant load running task at a speed 5% below the IAT (IAT – 5% of max. running velocity during incremental exercise test) with a prior 9 min running warm-up starting with 4–6 km/h and an incremental increase of running velocity every 3 min up to the constant load velocity (Moser et al., 2015). The IAT was used to determine the individual running velocity in each participant to account for different training levels and still ensure comparable but individual exercise intensity for a standardized time period. Blood lactate and heart rate were assessed at baseline and every 10 min during constant load running to control for steady state intensity. After running patients were asked to rate their perceived exertion using the Borg-scale (6–20) (Borg, 1982).

### Ultrasound Examinations

**FIGURE 3:**
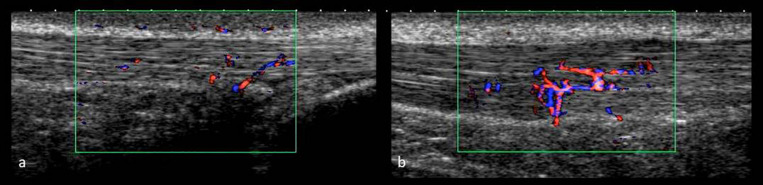
IBF images **(a)** Healthy, asymptomatic AT, and **(b)** Tendinopathic AT at U_5_.

All ultrasound examinations were performed by the same investigator applying a high-resolution ultrasound device (Xario SSA-660 A, Toshiba, Japan) with a multi-frequency linear transducer at 14 MHz (PLT-120 AT). Participants were examined in prone position with their feet hanging over the distal end of the examination table. On M1, tendon structure (hypo-/hyperechogenic alterations) and tendon anterior-posterior thickness at the thickest location (Fredberg et al., 2008) was assessed in longitudinal scans using B-mode (gain = 80, DR = 65, penetration depth = 3 cm, focus = 0.5 cm), the ankle being passively flexed at 90° angle to the tibia to avoid tendon waving (Fredberg et al., 2008). Doppler ultrasound examinations on M2 were performed with the feet hanging free and relaxed and applying minimal probe pressure to avoid obliteration of vessels (de Vos et al., 2007; Sengkerij et al., 2009; Cassel et al., 2018; Risch et al., 2018a). IBF was assessed with the broadband Doppler ultrasound “Advanced Dynamic Flow” (region of interest 2 × 1 cm, pre-settings: color gain 42, color velocity 1.5 cm/s, pulse repetition frequency 13.7 kHz) saving video sequences of 5 s as avi files (Figure 3). Each examination at U_pre_-U_120_ took approximately 5 min. At each measurement time point, ADF-scans were obtained from left and right AT in randomized order, assessing IBF throughout each tendon ranging from the calcaneal insertion to the musculo-tendinous junction of the soleus muscle. Tendons were scanned in medio-lateral direction gradually moving from distal to proximal, recording video sequences while following the course of intratendinous vessels whenever they were detected. IBF was quantified in dynamic scans by counting the total number of vessels [n] (every branch counting as one vessel) throughout each tendon (Risch et al., 2018a,b). The applicability as well as settings and procedure have been tested for validity and reliability in previous studies (Risch et al., 2016, 2018a,b). Doppler examination using “Advanced Dynamic Flow” has revealed high consistency compared with conventional Doppler ultrasound and good reliability of IBF assessment also for low level of observer experience (Risch et al., 2016). The grading procedure (counting number of vessels) has revealed high intra-observer test-retest agreement with a standard error of measurement of 0.99–1.47 vessels (Risch et al., 2018b).

### Data and Statistical Analysis

Anthropometric, exercise data, and tendon thickness measures are presented descriptively (mean ± SD). After testing for normal distribution (Shapiro-Wilk), group differences were analyzed using one-way ANOVA and Tukey-Kramer HSD *post hoc* test (SPSS Statistics 25 and Jmp 9.0). Since IBF data is not normally distributed within groups, IBF is presented descriptively (% of examined ATs, median and minimum-to-maximum range, and boxplots with interquartile range) for each group and measurement time point. Main effects for intra- and inter-group comparison were analyzed with Friedman and Kruskal-Wallis ANOVA (Wilcoxon test and Mann-Whitney-U test for *post hoc* analysis. Distribution of responding and non-responding ATs in the four groups was compared with contingency table and chi-square analysis. An association between amount of IBF at rest and amount of increase in U_5_ was analyzed using the Pearson correlation coefficient. Due to inter-individual variability of IBF amount no further statistical testing was performed. Significance level was set to α = 0.05.

After finding no statistically significant side differences between left and right tendon IBF amount in H_rec_ and H_run_, only right tendons are presented in comparison to the symptomatic tendon of T_rec_ and T_run_. In case of bilateral AT pathology in T_rec_ and T_run_, ATs with leading/more severe symptoms according to the clinical examination are considered the symptomatic side.

## Results

### Participants

There were no statistically significant group differences for age, height, and weight whereas kilometers of running per week, VISA-A, tendon thickness measures, and constant load velocity (M2) revealed significant differences between runners and recreational participants as well as between symptomatic and asymptomatic tendons (Table 1). Duration of symptoms in T_run_ and T_rec_ ranged from 2 months to more than 10 years (mean 26 months).

### Exercise Testing

M1 and M2 were separated 7 ± 6 days (2 to 16 days). On M2 all participants performed a constant load running task with a mean heart rate of 158 ± 13 and mean blood lactate of 2.7 ± 1.3 mmol. Subjective perceived exhaustion (Borg scale) after constant load running was reported to be 13 ± 1 in H_run_, 15 ± 2 in H_rec_, and 14 ± 2 in T_run_ as well as T_rec_ (*p* = 0.12).

### Intratendinous Blood Flow

At U_pre_, IBF was detected in 40% of H_run_ (median 3 vessels) and in 53% of H_rec_ (4 vessels). At U_5_ IBF responded to exercise in 30% of H_run_ (with a median difference to baseline of 3 vessels) and in 53% of H_rec_ (4 vessels) (Table 2). While the majority of responding ATs showed an exercise-induced increase, number of vessels temporarily decreased in two ATs. After 30 min, IBF returned to baseline values in 9 from 11 (80%) healthy responding ATs. A total of 60% of ATs in H_run_ and 33% in H_rec_ remained without any detectable IBF throughout the investigation. Within groups, changes over measurement time points were significant for H_rec_ (*p* < 0.01) between U_pre_ and U_5_ (*p* = 0.02) and between U_5_ and U_30_-U_120_ (*p* < 0.02). Exercise-induced changes of IBF in H_run_ did not reach statistical significance (*p* = 0.80) (Table 3). At all measurement time points, the amount of IBF between groups differed significantly (*p* < 0.05). IBF was significantly lower in H_run_ and H_rec_ compared to T_rec_ (*p* < 0.05) and in H_run_ compared to T_run_ (*p* = 0.04) at U_pre_-U_120_. Furthermore, H_rec_ was significantly lower than T_run_ at U_30_-U_60_ (*p* < 0.04). No significant differences were found between H_run_ and H_rec_ (*p* > 0.05) (Table 3).

**TABLE 2:** Descriptive data for IBF at baseline (Upre) and 5 min post-exercise (U5).

	Detectable IBF at Upre		Detectable IBF at U5	
		
Groups	Occurrence in % of ATs (x/n)	# of vessels median [range]	Responding ATs % (x/total n)	Median [range] diff. to Upre
Trun (*n* = 13)	85% (11/13)	3 (1 to 25)	70% (9/13)	4 (−10 to 10)
Trec (*n* = 10)	70% (7/10)	10 (2 to 30)	80% (8/10)	5 (1 to 10)
Hrun (*n* = 10)	40% (4/10)	3 (1 to 6)	30% (3/10)	3 (−1 to 9)
Hrec (*n* = 15)	53% (8/15)	4 (1 to 5)	53% (8/15)	4 (−2 to 6)

*Presented data only refer to ATs with detectable IBF.*

**TABLE 3:** IBF at each measurement time point (U5-U120) median [range] (value represent median for whole group).

Groups	Measurement time point					*p-value*
		
	Upre	U5	U30	U60	U120	(*within group*)
Trun	3 (0–25)^k^	7 (0–19)^k^	4 (0–23)^km^	4 (0–26)^km^	3 (0–25)^km^	*0.05*
Trec	8 (0–30)^agh^	12 (0–40)^agh^	9 (0–40)^gh^	9 (0–30)^gh^	9 (0–30)^gh^	<*0.01*
Hrun	0 (0–6)^hk^	0 (0–10)^hk^	0 (0–7)^hk^	0 (0–6)^hk^	0 (0–6)^hk^	*0.80*
Hrec	1 (0–5)^adg^	4 (0–11)^abcdfg^	2 (0–7)^bdgm^	2 (0–7) ^cdgm^	2 (0–6)^fgm^	<*0.01*
*p-value (between groups)*	*0.04*	*0.02*	*0.02*	*0.03*	*0.03*	

*All values refer to total group; ^*a b c d*^ indicates sig. difference in *post hoc* analysis within group (*p* < 0.02); ^*g h k m*^ indicate sig. difference in *post hoc* analysis between groups (*p* < 0.05).*

In tendinopathic participants, baseline examination showed IBF in 85% of T_run_ (3 vessels), and in 70% of T_rec_ (10 vessels). At U_5_ IBF responded to exercise in 70% of T_run_ (with a median difference to baseline of 4 vessels) and in 80% of T_rec_ (5 vessels) (Table 2). One from 17 responding ATs showed a temporary reduction of IBF at U_5_. In 10 from 17 (60%) responding tendinopathic ATs IBF remained increased after U_30_. Some ATs remained without detectable IBF throughout the investigation (15% in T_run_ and 8% in T_rec_). Within groups, IBF changes over time were significant for T_run_ (*p* = 0.05) and T_rec_ (*p* < 0.01). *Post hoc* analysis showed statistically significant differences only between U_pre_ and U_5_ for T_rec_ (*p* = 0.01) but not for T_run_ (*p* = 0.08). No significant between group differences were detected for T_run_ and T_rec_ at any measurement time point (*p* > 0.05).

**FIGURE 4:**
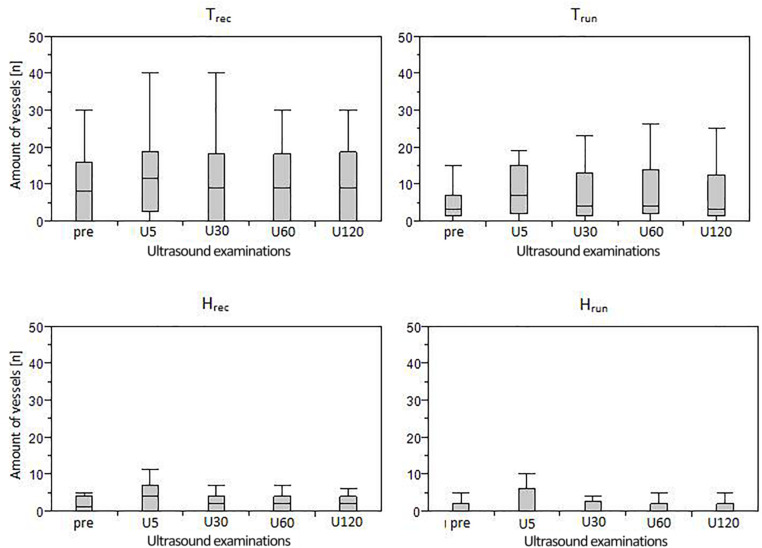
Boxplots (median and interquartile range) of IBF amount in each group assessed during five measurement time points (U_pre_-U_120_).

**FIGURE 5:**
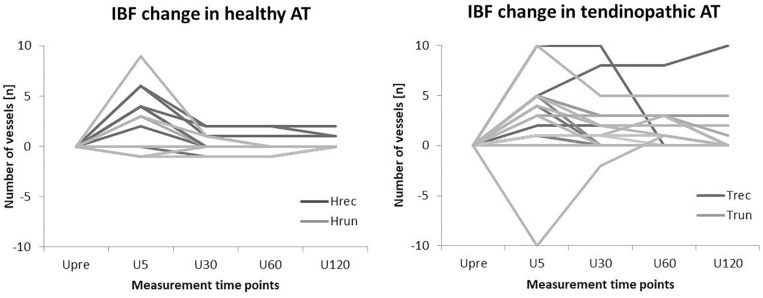
Individual courses of IBF changes relative to U_pre_ in healthy and tendinopathic ATs.

The distribution of ATs responding and not responding to exercise did not differ significantly between groups (*p* = 0.11). Furthermore, the amount of IBF increase was not significantly correlated to the amount of IBF at baseline (*p* = 0.56). Boxplots with median number of vessels and interquartile range for each group (in total) and measurement time point is presented in Figure 4. Individual courses of IBF change relative to baseline in healthy and tendinopathic Achilles tendons is presented in Figure 5.

## Discussion

This study investigated the acute effect of constant load running on presence of sonographic detectable IBF in runners and recreational participants with and without tendon pathology. Irrespective of training and pathology, some ATs responded with acutely increased IBF 5 min after exercise while others remained unaffected. The amount of IBF increase was similar (median of 3–5 vessels) in all four groups. The immediate increase, however, only reached the level of significance in H_rec_ and T_rec_. Contrary to the hypothesis of a pronounced reaction in athletes, runners neither revealed a significantly higher amount of IBF at rest nor following exercise compared to recreational participants. Although healthy and tendinopathic ATs showed a similar IBF increase directly after exercise, the overall amount of intratendinous vessels was significantly higher in tendinopathic compared to healthy ATs at all measurement time points. Moreover, the duration of increased IBF after exercise differed between healthy and tendinpathic ATs. While IBF increase in H_rec_ was only detectable 5 min after exercise and returned to baseline values within 30 min, IBF increase detected in T_rec_ did not show a significant decrease at U_30_-U_120_.

The role of exercise-induced IBF increase in tendinopathy patients has been discussed controversially. On the one hand, IBF was assumed to represent a pathological finding and exercise was necessary to reveal total amount of vascularity (Cook et al., 2005). On the other hand, it has been discussed to be a physiological reaction associated to increased metabolic demands (e.g., oxygen demand and collagen synthesis) (Boushel et al., 2000; Kjaer et al., 2005; Boesen et al., 2006b). While an acute short-term increase directly following exercise has been considered to represent a physiological effect, a prolonged elevation seen in 60% of responding tendinopathic ATs, is hypothesized to be an indicator of morphological changes and tissue turnover and might represent a possible discrimination factor between healthy and pathological ATs. In this context, a post-exercise Doppler ultrasound examinations performed after 30 min of rest would exclude physiological exercise-induced IBF (Risch et al., 2018a). However, since the observation of a prolonged IBF response did not reach statistical significance, it requires confirmation in further investigations with a larger sample size.

Irrespective of exercise, between group comparison showed a significantly higher IBF level in tendinopathic compared to healthy ATs, which is similar to findings from Boesen et al. (2006b) and Pingel et al. (2013a). In patients with Achilles tendinopathy, occurrence of resting IBF (2 to 30 vessels) in 70–85% of ATs is in line with previous reports ranging from 50 to 88% (Tol et al., 2012). However, prospective studies have shown that the presence and amount of IBF as well as structural alterations of tendon tissue in tendinopathy patients are not necessarily associated with the degree of tendon pain and functional impairment (de Vos et al., 2007; De Jonge et al., 2014; Ryan and Cped, 2015). IBF is rather seen as part of a failed tendon healing process including matrix change and ingrowth of sensory and sympathetic nerves (Andersson et al., 2007; Tol et al., 2012). Although degenerated tendons frequently reveal higher amount of IBF at rest, examinations using microdialysis have reported high lactate levels suggesting an anaerobic metabolism in a persisting hypoxic environment (Alfredson et al., 2002; Järvinen, 2020). Therefore, Järvinen (2020) has proposed that sonographic detectable intratendinous vessels in degenerated tendons represent hypoxia-induced, non-functional neo-vessels that fail to supply tendon tissue with oxygen and nutrients required for tissue healing. On the other hand, some papers have discussed IBF to be a positive finding indicating a reparative healing response e.g., following eccentric training and sclerosing injections, which remains to be clarified in further studies (Alfredson and Ohberg, 2006; Pingel et al., 2013a; De Marchi et al., 2018).

In healthy athletes, increased amount of resting IBF has been proposed to represent a long-term physiological metabolic adaptation to prolonged sports performance involving high and repetitive mechanical loading (Boesen et al., 2006a, 2011, 2012; Malliaras et al., 2008; Koenig et al., 2010). It has been suggested that this elevated vascularization indicates a continuous regenerative process during resting phases in between training sessions (Boesen et al., 2006a). Investigations regarding the acute effect of exercise in athletes have reported persisting or increased IBF following sport performance in elite badminton players (Boesen et al., 2006a, 2011) and recreational floorball players (Fahlström and Alfredson, 2010), assuming a physiological remodeling process and reaction to hypoxic environment in direct response to tendon loading (Koenig et al., 2010; Boesen et al., 2011; Malliaras et al., 2012). On the other hand, IBF was discussed to indicate overloading and pathology (Ohberg et al., 2001; Boesen et al., 2006a, 2011) which would implicate a number of asymptomatic athletes with pathological tendons (Boesen et al., 2006a, 2011; Tardioli et al., 2012). Since the present study does not show significantly altered resting IBF or pronounced exercise-induced IBF increase in runners compared to recreational participants, the previously suggested long-term adaptation regarding increased detectability of IBF in athletes (Malliaras et al., 2008; Boesen et al., 2012) cannot be supported.

Temporary increased IBF solely detectable directly following tendon loading exercise may be considered a physiological response irrespective of training status or tendon pathology. A comparable acute response to exercise has already been reported in a prior pilot study with healthy recreational participants (Risch et al., 2018a) as well as by Boesen et al. (2006b) finding resting-IBF in 30% (“mean color fraction” 0.05) of ATs in 10 healthy non-trained participants and increased or sustained IBF in 80% (mean color fraction 0.14) after a 5 km run. Furthermore, elevated IBF reported in elite badminton players (Boesen et al., 2006a) and recreational floorball players (Fahlström and Alfredson, 2010) directly after competition and in Achilles tendinopathy patients directly after eccentric training (Boesen et al., 2006b) support this assumption. It is also known from microdialysis, Xenon clearance technique, and contrast-enhanced ultrasound examinations that physiological tendon blood flow can increase up to 12-fold following tendon loading exercise (Kjaer et al., 2006; Pingel et al., 2013a,b). Therefore, detectability of exercise-induced IBF increase in the present study is assumed to represent a physiologically increased flow in preexisting vessel beds temporarily rising above the sensitivity threshold of the applied ultrasound device (Boesen et al., 2006b; Risch et al., 2018a). Present and previous findings indicate that there are “responders” and “non-responders” regarding sonographic detectable IBF changes following acute exercise. Although the percentage of responders is lower in healthy compared to tendinopathic ATs, statistical analysis revealed no significant differences between groups. If varying responsiveness regarding the sonographic detectable increase of IBF is a consequence of inter-individual differences in tendon vascularization (Boesen et al., 2006b; Risch et al., 2018a) can only be speculated.

In healthy ATs the occurrence of 1 to 6 vessels in 40–53% of ATs was higher than the previously reported detectability in 29–35% of ATs in comparable participants (Boesen et al., 2006b; Hirschmüller et al., 2010; Risch et al., 2018a). Boesen et al. (2006b, 2011) have suggested that low amount of sonographic detected resting IBF (grade 1 with 1 or 2 “small color foci”) represents a physiological finding whereas higher IBF amount was considered pathological. A specific threshold between pathological and physiological IBF, however, has not been established yet (Boesen et al., 2006b, 2011, 2012; Koenig et al., 2010; Genovese et al., 2011). On the other hand, it is known that tendon blood flow commonly exists below the sensitivity threshold of conventional Doppler ultrasound devices (Tol et al., 2012; Pingel et al., 2013b). Improving technology with increased sensitivity, e.g., use of Doppler ultrasound mode “Advanced Dynamic Flow”(Risch et al., 2016), has likely resulted in detection of physiological, low vascularization (Boesen et al., 2012; Tol et al., 2012) not visible in earlier studies (Ohberg et al., 2001). For the interpretation of detectable IBF, presence of pain and tendon tissue alterations should be taken into account. Data of the present study suggest that in the absence of tendon pain in history, pain on palpation and sonographic detectable pathology, the observed 6 vessels at rest and an increase up to 10 vessels following exercise assessed with “Advanced Dynamic Flow” can be considered a physiological finding. The determination of a specific threshold for physiological versus pathological IBF, however, requires further investigation and is likely to be device-dependent (Boesen et al., 2006b).

### Limitations

To ensure comparable intensity of cardiovascular, metabolic and mechanical stress, the constant load running exercise was standardized based on running velocity and exercise duration, taking into account that the distance covered varied between individual participants. Standardized device settings, reliability of measurement procedure and data analysis have been tested previously (Risch et al., 2016, 2018a,b). To optimize reliability, all examinations have been performed by the same experienced investigator (Risch et al., 2016, 2018b). It was taken into account that due to the clinical setting of this study, the examiner was not blinded to the participant and measurement time point. Nevertheless, some consecutive post-exercise Doppler ultrasound examinations in H_rec_ and H_run_ revealed slight variations of one vessel which were most likely due to ultrasound handling and are considered negligible due to measurement error. A temporary reduction of 10 vessels in one tendon of the T_run_ group at U_5_, however, was remarkable. Other studies have reported this occurrence in some tendons as well but had no conclusive explanation either (Boesen et al., 2006b; Malliaras et al., 2012).

Due to a lack of comparable and available data, no power-analysis was performed prior to this study. Therefore the aim was to include a minimum of 10 participants per group, comparable to sample sizes in previous studies investigating exercise-induced IBF (Boesen et al., 2006b; Malliaras et al., 2012; Pingel et al., 2013b), with an age and gender matched composition. The relatively small sample size may influence the generalizability of the presented results and main effects. Furthermore, the inter-subject variability concerning amount of IBF as well as supposed presence of responders and non-responders to exercise limit the ability to perform statistical testing: Due to lack of normal distribution, statistical analysis was performed using a non-parametric ANOVA which does not test for an interaction effect. Nevertheless, the described varying IBF occurrence is comparable to findings in previous literature (Hirschmüller et al., 2010; Risch et al., 2016). Previous investigations applying conventional Doppler ultrasound commonly involve rather imprecise and “noisy” depiction of IBF due to the so-called “blooming effect” (Heling et al., 2004). The use of the ADF-mode with highly standardized assessment settings has enabled a very detailed and reliable imaging and quantification of IBF. Therefore, the results are considered clinically relevant. However, it should be noted that the response of IBF presented in this study merely refers to sonographic detectable blood flow and excludes changes in microvascular volume existing below the sensitivity threshold of the applied Doppler mode.

## Conclusion

In conclusion, this study showed that exercise predominantly results in a slight immediate increase of sonographic detectable IBF irrespective of training status and tendon pathology. In all groups there are responders and non-responders to tendon loading activity regarding changes of IBF. It is not feasible to discriminate between IBF in runners and recreational participants since runners did not show an elevated amount of resting-IBF or a pronounced response following exercise. Although tendinopathic ATs reveal an overall higher level of IBF compared to healthy ATs at all times, the amount of increase following exercise was similar. While a temporary exercise-induced IBF increase solely detectable after 5 min is considered a physiological response, prolonged IBF elevation may indicate morphological changes and tendon tissue turnover which has to be confirmed in further studies. In healthy ATs, the present study found presence of up to 6 vessels at rest and up to 10 vessels following exercise which is considered a physiological finding. However, the determination of a specific threshold to differentiate between physiological (low) and pathological (high) IBF requires further investigation.

## Data Availability Statement

The raw data supporting the conclusions of this article will be made available by the authors, without undue reservation.

## Ethics Statement

The studies involving human participants were reviewed and approved by the Ethics Committee of the University of Potsdam. The patients/participants provided their written informed consent to participate in this study.

## Author Contributions

LR, FM, and MC involved in the development of study design, interpretation, and revising the manuscript. LR involved in the data collection, data analysis, and preparation of the manuscript. All authors contributed to the article and approved the submitted version.

## Conflict of Interest

The authors declare that the research was conducted in the absence of any commercial or financial relationships that could be construed as a potential conflict of interest.
